# Self-transfers and factors associated with successful tracing among persons lost to follow-up from HIV care, Sheema District, Southwestern Uganda: retrospective medical records review, 2017–2021

**DOI:** 10.1186/s12981-022-00471-2

**Published:** 2022-09-20

**Authors:** Arnold Ssemwogerere, Javilla Kakooza Kamya, Lillian Nuwasasira, Claire Ahura, Derrick Isaac Isooba, Edith K. Wakida, Celestino Obua, Richard Migisha

**Affiliations:** 1 Department of Pharmacy, Mbarara University of Science and Technology, Mbarara, Uganda; 2 Department of Internal Medicine, Mbarara University of Science and Technology, Mbarara, Uganda; 3 Department of Pharmaceutical Sciences, Mbarara University of Science and Technology, Mbarara, Uganda; 4 Department of Nursing, Mbarara University of Science and Technology, Mbarara, Uganda; 5 Department of Medical Laboratory Sciences, Mbarara University of Science and Technology, Mbarara, Uganda; 6 Department of Medical Education, California University of Science and Medicine, San Bernardino, USA; 7 Office of Research Administration, Mbarara University of Science and Technology, Mbarara, Uganda; 8 Office of the Vice Chancellor, Mbarara University of Science and Technology, Mbarara, Uganda; 9 Department of Physiology, Mbarara University of Science and Technology, P.O BOX 1410, Mbarara, Uganda

**Keywords:** Loss to follow-up, Antiretroviral therapy, HIV, Self-transfer, Tracing, Uganda

## Abstract

**Background:**

Due to improved coverage and scale-up of antiretroviral therapy (ART), patients are increasingly transferring between ART-providing sites. Self-transfers may constitute a high proportion of patients considered lost to follow-up (LTFU), and if overlooked when reporting patients who have dropped out of HIV care, may result in an incorrect estimation of retention. We determined the prevalence of self-transfers, and successful tracing, and identified associated factors among people living with HIV (PLHIV) LTFU from care at public health facilities in Sheema District, Southwestern Uganda.

**Methods:**

We conducted a cross-sectional retrospective medical records review during February and March 2022. We included records of all PLHIV who were LTFU from 2017 to 2021, and who were registered at government-owned ART clinics in Sheema District. LTFU was considered for those who were not taking ART refills for a period of ≥ 3 months. We abstracted demographic and clinical data from medical records at the selected clinics. Participants were traced via phone calls or in-person to ascertain the outcomes of LTFU. We performed multivariate modified Poisson regression to identify factors associated with self-transfer, and successful tracing.

**Results:**

Overall, 740 patients were identified as LTFU from three ART-providing clinics; of these, 560 (76%) were self-transfers. The mean age was 30 (SD ± 10) years, and most (69%, n = 514) were female; the majority (87%, 641/740) were successfully traced. Age (adjusted prevalence ratio [aPR] = 1.13, 95% CI 1.01–1.25, *P* = 0.026 for those aged 18–30 years compared to > 30 years), female sex (aPR = 1.18, 95% CI 1.11–1.25, *P* < 0.001), and having WHO clinical stage 1–2 (aPR = 2.34, 95% CI 1.89–3.91, *P* < 0.001) were significantly associated with self-transfer. Presence of a phone contact in the patient’s file (aPR = 1.10, 95% CI 1.01–1.90, *P* = 0.026) was associated with successful tracing of the patients considered LTFU.

**Conclusion:**

Self-transfers accounted for the majority of patients recorded as LTFU, highlighting the need to account for self-transfers among patients considered LTFU, to accurately estimate retention in care. ART-providing facilities should regularly update contact information for PLHIV to enable successful tracing, in the event that the patients are LTFU. This calls for a health-tracking system that easily identifies self-transfers across ART-providing clinics using unique patient identifiers.

## Introduction

Globally, approximately 37.7 million individuals had HIV infection in 2020, with 27.5 million having access to antiretroviral therapy (ART) [1]. Globally, by the end of 2020, 84% of people living with HIV (PLHIV) knew their HIV status, 87% of those who knew their HIV status were on ART, and 90 percent of those on ART had achieved viral load suppression [1]. In Uganda, as of 2019, 90 percent of PLHIV were aware of their HIV status, 96 percent of those who tested positive were on ART, and 87 percent had achieved viral suppression[2]. Accordingly, significant progress has been made toward meeting the 90–90–90 targets, which were only narrowly missed in 2019.

Since the World Health Organization (WHO) issued the “treat all” guidelines in 2015 [3], the number of persons initiated on ART has steadily increased in sub-Saharan Africa (SSA). By 2020, 19.4 million individuals in SSA had been initiated on ART [1]. Despite the fact that access to ART has increased significantly in SSA, ART program success is dependent on retention in care. One of the many issues that can affect HIV care retention is loss to follow-up. In SSA, where there is a high burden of HIV, approximately one-third of the PLHIV are lost to follow-up (LTFU) within three years of starting ART [4].

In November 2016, Uganda formally implemented the “treat all” guidelines, under which ART was provided to all HIV-positive persons regardless of CD4 count or clinical stage. By the end of 2017, nearly all ART-providing health facilities in Uganda had implemented the guidelines [5]. As a result, many individuals who are enrolled in HIV care are asymptomatic, and therefore are likely to present different challenges with regard to retention in care. Some authors hypothesized that most of the losses from ART clinics after the first year of the “treat all” guidelines’ implementation would be unreported transfers to new ART-providing sites [6]. In addition, it has been observed that as ART coverage and decentralization of ART services to primary care expand, patients are increasingly transferring between ART clinics [7]. These transfers, if undocumented, are referred to as ‘self-transfers’. Self-transfers may be overlooked when reporting patients who have dropped out of HIV care, resulting in an incorrect estimation of retention. In the current setting of rising ART program expenses and limited donor financing, accurate estimates of retention in care are more crucial than ever, to accurately forecast utilization of HIV-related medications and other supplies.

The Joint UN Program on HIV/AIDS (UNAIDS) recently issued the “95–95–95” targets with the aim of ending the AIDS epidemic by 2030 [8]. To meet this ambitious target of ending the HIV pandemic, it is necessary to trace patients considered LTFU, and reengage them to optimize their HIV care environment [9]. Data on successful tracing of patients LTFU in Uganda are limited. Prior tracing studies conducted in Uganda, focused on correction of estimates of retention in care in Central Uganda [5, 10]. Additionally, the previous studies did not focus on self-transfers. Nonetheless, successful tracing of patients considered LTFU in resource-limited settings, such as Uganda, may be challenging due to a lack of proper documentation systems, and inadequate staffing. The primary aim of this study was to determine the prevalence of self-transfers and associated factors among PLHIV LTFU from care at public health facilities in Sheema District, Southwestern Uganda from 2017 to 2021.The secondary aim of the study was to identify factors associated with successful tracing of PLHIV recorded as LTFU from the public health facilities in Sheema District from 2017 to 2021.

## Methods

### Study design and setting

We conducted a cross-sectional retrospective medical records review and phone interviews with PLHIV LTFU or their next of kin, during February and March 2022. The study utilized data of PLHIV, who were registered at the ART clinics in Sheema District, Southwestern Uganda. The district has prevalence of HIV of 7.9%. Sheema has an estimated catchment population of approximately 800,000 persons. The study was conducted in three purposively-selected ART clinics, which are government-owned health facilities namely: Kitagata Hospital, Shuuku Health Centre IV, and Kabwohe Health centre IV. The health facilities were purposively selected because they are high-level facilities with large volume clinics, attending to > 600 PLHIV (from a catchment population of ≥ 100,000 per health facility) [11]. The ART clinics at Shuuku and Kabwohe HCIVs operate 2–3 days per week, while the ART clinic at Kitagata Hospital operates four days per week. At all the three health facilities, the patient transfer process begins with the patient approaching the head of the ART clinic and explaining why he or she is transferring. A health worker then completes a transfer form with the patient’s details, including file number, CD4 count, regimen details, and current viral load. The patient is then given a transfer letter that introduces him or her to the new ART clinic where he or she will be treated. However, the vast majority of patients self-refer to other ART facilities without following this procedure.

### Population and sample size

We studied all 740 PLHIV who were identified as LTFU from 2017 to 2021 at the selected government-owned ART clinics in Sheema District, Southwestern Uganda. The definition of LTFU used in this study was adopted from the Uganda Ministry of Health guidelines; accordingly, LTFU was considered for those who were not taking an ART refill for a period of ≥ 3 consecutive months [12].

### Data collection procedures, and study variables

Data abstraction form was used to extract data from patients’ records at the selected health facilities. Additionally, patients identified as LTFU were contacted through phone calls using contact information obtained from the patients’ medical records, to supplement on data obtained from the medical records. The patient contact information included their phone number and that of their next of kin, and the physical address. Prior to conducting telephone interviews, the health workers explained the purpose of the study, and obtained verbal informed consent from the patients, or their next of kin. Additional data extracted from the medical records included: socio-demographics (age, sex, marital status, level of education), date of enrolment into care, and date of loss to follow-up from care. Laboratory data included viral load before loss to follow-up. Unsuppressed viral load was defined as viral load of > 1000 copies/ml [12]. Data obtained from the patient or their next of kin/family member through phone-call interviews included: outcomes of loss to follow-up (whether patients died, withdrew from care, self-transferred, or restarted ART) and reasons for self-transfer or withdrawal from care.

Data were collected by the health workers (nurses) who work in the respective ART clinics in the selected health facilities. Prior to data collection, the nurses were trained on the research protocol and data collection tool and procedures. All patients' files at the ART facilities were reviewed by the data collectors to ascertain those who had been identified as LTFU from 2017 to 2021. Tracing was done by the healthcare workers at the respective ART clinics, who contacted the patients or family members, or contact persons, by phone or in person. Participants who were traced in-person provided written informed consent, while those who were traced via phone calls provided verbal informed consent, prior to participation, as approved by the Mbarara University of Science and Technology Research Ethics Committee (MUST-REC), under registration number, MUST-2021-289. Tracing was considered successful for those LTFU participants who were able to be contacted or their next of kin (via phone calls or in-person) to determine their outcome of being LTFU from HIV care.

### Data management and statistical analysis

The data were entered in Epi Info version 7 (CDC, Atlanta, USA), and all statistical analyses were done using Stata Version 15 (StataCorp, Texas, USA). The prevalence of self-transfer was determined as a proportion of patients LTFU, who transferred to other ART-providing sites without documentation. Socio-demographic and clinical characteristics of the study participants were presented as frequencies and percentages, for categorical variables. Continuous normally distributed variables such as age, were presented as means (± standard deviation [SD]), while non-normally distributed variables (e.g., duration in care before loss to follow-up) were summarized as median (interquartile range [IQR]). Our outcome variables of interest were self-transfer and successful tracing of participants LTFU. We used modified Poisson regression to fit univariate and multivariate multi-level mixed effects generalized linear models to identify factors associated with self-transfer, and successful tracing. We reported prevalence ratios as our measures of association. Due to the high prevalence of our outcome variables (self-transfer and successful tracing), we chose modified Poisson regression over logistic regression, because odds ratios would potentially overestimate the effect size [13]. We included variables as categorical fixed effects nested within fixed health facility identifiers to account for clustering of observations at the different ART-providing health facilities, and we assumed normal distribution of the random effects. Variables with p-value < 0.2 in univariate analyses were entered to multivariate models to obtain adjusted prevalence ratios that were mutually adjusted for all other predictor variables. We included sex and age in our final model because they are known confounders [14]. The adjusted prevalence ratios with p-value ≤ 0.05 were considered statistically significant.

## Results

### Characteristics of study participants

The mean age of study participants who were identified as LTFU was 30 (SD ± 10) years, with most participants in the age group of 18–30 years (57%, 424/740). Majority were female (69%, 510/740), had received formal education (88%, 648/740), had WHO clinical stage 1 (91%, 675/740), and had their viral loads suppressed or undetectable (85%, 632/740), as shown in Table 1. Similarly, among the 560 participants who self-transferred, most were aged 18–30 years (61%, n = 342), were female (74%, n = 415), had received formal education (88%, n = 492), had their viral loads suppressed or undetectable and had WHO clinical stage 1 (85%, n = 478) (Table 1).

**Table 1:** Characteristics of participants who were lost to follow-up, successfully traced, and self-transferred from public health facilities, Sheema District, 2017–2021

Characteristic	Total Lost to follow-up (N = 740)		Self-transferred (n = 560)		Successfully traced (n = 641)	
	n	(%)	n	(%)	n	(%)
Age^a^ in years						
< 18 years	19	(3)	16	(3)	18	(3)
18–30 years	424	(57)	342	(61)	355	(55)
> 30 years	297	(40)	202	(36)	268	(42)
Sex						
Female	510	(69)	415	(74)	442	(69)
Male	230	(31)	145	(26)	199	(31)
Marital status						
Single	199	(27)	153	(27)	170	(27)
Married	410	(55)	321	(57)	353	(55)
Divorced/separated	103	(14)	70	(13)	91	(14)
Widowed	28	(4)	16	(3)	27	(4)
Received formal education						
No	92	(12)	68	(12)	80	(12)
Yes	648	(88)	492	(88)	561	(88)
Duration in care before LTFU						
< 1 year	295	(40)	229	(41)	257	(40)
1–5 years	366	(49)	283	(50)	313	(49)
> 5 years	79	(11)	48	(9)	71	(11)
HIV clinical stage before LTFU^b^						
One	675	(91)	532	(95)	586	(91)
Two	31	(4)	18	(3)	27	(4)
Three	28	(4)	9	(2)	23	(4)
Four	6	(1)	1	(0)	5	(1)
Viral load before LTFU						
Suppressed or undetectable	632	(85)	478	(85)	542	(85)
Unsuppressed	108	(15)	82	(15)	99	(15)
Client phone number available						
No	535	(72)	384	(69)	452	(71)
Yes	205	(28)	176	(31)	189	(29)
Client physical address available						
No	331	(45)	266	(47)	295	(46)
Yes	409	(55)	294	(53)	436	(54)
Next of kin phone number available						
No	612	(83)	467	(83)	526	(82)
Yes	128	(17)	93	(17)	115	(18)

LTFU, loss to follow-up

^a^Mean age = 30 (SD ± 10) years, median age = 28 (IQR = 23–35) years

^**b**^Median duration = 1 year (IQR: 3 months–3 years)

Among the those who were successfully traced, more than half (55%, 355/641) were aged 18–30 years, the majority were female (69%, 442/641), had received formal education (88%, 561/641), had WHO clinical stage 1 (91%, 586/641), and had their viral loads suppressed or undetectable (85%, 542/641) (Table 1).

### Prevalence of self-transfers and successful tracing among participants lost to follow-up

Of the 740 patients LTFU, 560 (76%; 95% CI 72–79%) were self-transfers, 37 (5%) had withdrawn from care, 14 (1.9%) had been restarted on ART, 93 (13%) had died, and the remaining 36 (4.9%) had unknown outcome. Overall, 87% (n = 641; 95% CI 84–89%) were successfully traced. Of the 37 participants who withdrew from care, most were aged 18–30 years (62%, n = 23), had WHO clinical stage 1 (89%, n = 33), and were female (61%, n = 23).

### Reasons for self-transferring

**Fig. 1:**
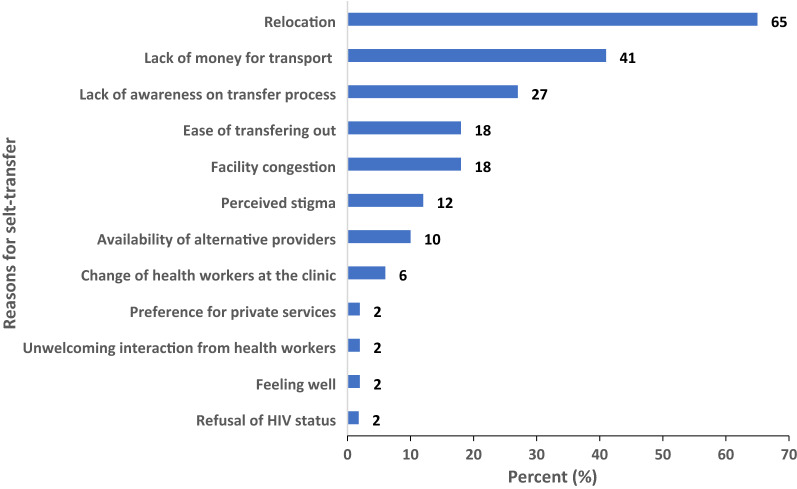
Reasons for self-transfer among successfully traced patients lost to follow-up (n = 493) in public health facilities, Sheema District, Southwestern Uganda, 2017–2021

Of the 560 patients who self-transferred, 493 (88%) were successfully reached through phone calls, most of whom reported that relocation (65%, n = 320) and lack of transport (41%, n = 202) were the main reasons for self-transfer (Fig. 1). Slightly over one-quarter (27%, n = 133) of the those successfully traced reported lack of awareness on the transfer process.

The distribution of demographic and clinical characteristics of the self-transfers who were interviewed for the reasons for self-transfer and those who were not reached via phone calls was not statistically different between the two groups (Table 2).

**Table 2:** Comparison of clinical and demographic characteristics of self-transfers who were interviewed via phone calls and those who were not interviewed

Variable	Self-transfer (n = 560)		P-value
	interviewed (n = 493), n (%)	Not interviewed (n = 67), n (%)	
Age in years			0.462
< 18 years	15 (3)	1 (2)	
18–30 years	299 (61)	43 (64)	
> 30 years	179 (36)	23 (34)	
Sex			0.639
Female	362 (73)	51 (76)	
Male	131 (27)	16 (24)	
Marital status			0.057
Single	130 (26)	23 (34)	
Married	282 (57)	39 (58)	
Divorced/separated	66 (13)	4 (6)	
Widowed	15 (3)	1 (2)	
Duration in care before LTFU			0.890
< 1 year	202 (41)	27 (40)	
1–5 years	249 (51)	34 (51)	
> 5 years	42 (9)	6 (9)	
HIV clinical stage before LTFU			0.092
One	465 (95)	63 (94)	
Two	17 (4)	1 (2)	
Three or Four	7 (1)	3 (5)	
Viral load			0.506
Suppressed or undetectable	419 (85)	59 (88)	
Unsuppressed	82 (15)	8 (12)	

LTFU, loss to follow up

In the multivariate analysis (Table 3), age, sex and HIV clinical stage before LTFU were the factors significantly associated with self-transfer. The prevalence of self-transfer was 13% higher (aPR = 1.13, 95% CI 1.01–1.25, *P* = 0.026) among participants aged 18–30 years, compared to those aged > 30 years. The prevalence of self-transfer was 18% higher (aPR = 1.18, 95% CI 1.11–1.25, *P* ≤ 0.001) among females compared to males, and 2.3 times higher (aPR = 2.34, 95% CI 1.89–3.91, *P* < 0.001) among participants with asymptomatic or mild disease (WHO clinical stage 1–3) compared to others.

**Table 3:** Factors associated with self-transfer among participants lost to follow-up from public health facilities, Sheema District, Southwestern Uganda, 2017–2021

Variable	Self-transferred		Univariate analysis		Multivariate analysis	
	Yes (n = 560), n (%)	No (n = 180), n (%)	cPR (95% CI)	P value	aPR (95% CI)	P value
Age in years						
< 18 years	16 (3)	3 (2)	1.12 (0.95–1.68)	0.112	1.20 (0.92–1.55)	0.174
18–30 years	342 (61)	82 (46)	1.19 (1.08–1.33)	0.002	1.13 (1.01–1.25)	0.026
> 30 years	202 (36)	95 (53)	Ref		Ref	
Sex						
Female	415 (74)	99 (55)	1.23 (1.17–1.29)	< 0.001	1.18 (1.11–1.25)	< 0.001
Male	145 (26)	81 (45)	Ref		Ref	
Received formal education						
No	68 (12)	24 (13)	Ref			
Yes	492 (88)	156 (87)	1.06 (0.94–1.20)	0.317		
Marital status						
Single	153 (27)	46 (26)	Ref			
Married	321 (57)	89 (49)	1.12 (0.93–1.37)	0.233		
Divorced/separated	70 (13)	33 (18)	1.12(0.88–1.42)	0.375		
Widowed	16 (3)	12 (7)	0.90 (0.75–1.09)	0.294		
Duration in care before LTFU						
< 1 year	229 (41)	66 (37)	Ref			
1–5 years	283 (50)	83 (46)	1.02 (0.85–1.23)	0.822		
> 5 years	48 (9)	31 (17)	0.79 (0.52–1.22)	0.291		
HIV clinical stage before LTFU						
Stage 1–2	550 (98)	156 (87)	2.44 (1.96–3.04)	< 0.001 s	2.34 (1.89–3.91)	< 0.001
Stage 3–4	10 (2)	24 (13)	Ref		Ref	
Viral load						
Suppressed or undetectable	478 (85)	154 (86)	Ref			
Unsuppressed	82 (15)	26 (14)	0.97 (0.89–1.05)	0.454		

cPR, crude prevalence ratio; aPR, adjusted prevalence ratio; Ref, reference category, LTFU, loss to follow-up; CI, confidence interval

### Factors associated with successful tracing of participants lost to follow-up

At multivariate analysis (Table 4), the only factor that was significantly associated with successful tracing of participants LTFU was presence of phone contact for the patient on their medical records. Patients who had their phone numbers recorded in their files had 10% higher prevalence (aPR = 1.10; 95% CI 1.01–1.90, *P* = 0.026) of being successfully traced compared to their counterparts (Table 4).

**Table 4:** Factors associated with successful tracing of participants lost to follow-up from public health facilities, Sheema District, Southwestern Uganda, 2017–2021

Variable	Successfully traced		Univariate analysis		Multivariate analysis	
	Yes (n = 641), n (%)	No (n = 99), n (%)	cPR (95% CI)	P value	aPR (95% CI)	P value
Age in years						
< 18 years	18 (2.8)	1 (1.0)	1.12 (0.95–1.37)	0.160	1.14 (0.94–1.39)	0.173
18–30 years	355 (55)	69 (70)	Ref		Ref	
> 30 years	268 (42)	29 (29)	1.08 (0.95–1.22)	0.226	1.08 (0.95–1.24)	0.253
Sex						
Female	442 (69)	72 (73)	Ref		Ref	
Male	199 (31)	27 (27)	1.03 (0.95–1.12)	0.510	1.00 (0.91–1.11)	0.970
Received formal education						
No	80 (12)	12 (12)	Ref			
Yes	561 (88)	87 (88)	0.99 (0.87–1.13)	0.868		
Marital status						
Single	170 (27)	29 (30)	Ref		Ref	
Married	282 (57)	39 (58)	0.97 (0.86–1.10)	0.674	0.98 (0.87–1.11)	0.771
Divorced/separated	91 (14)	12 (12)	0.97(0.96–1.02)	0.199	0.98 (0.93–1.04)	0.494
Widowed	27 (4.2)	1 (1.0)	1.10 (1.03–1.16)	0.003	1.08 (0.98–1.12)	0.056
Duration in care before LTFU						
< 1 year	257 (40)	38 (38)	Ref		Ref	
1–5 years	313 (49)	53 (54)	1.02 (0.97–1.08)	0.380	1.04 (0.96–1.14)	0.348
> 5 years	71 (11)	8 (8.1)	1.05 (1.01–1.10)	0.026	1.03 (0.94–1.11)	0.366
HIV clinical stage before LTFU						
Stage 1–2	613 (96)	93 (94)	Ref		Ref	
Stage 3–4	28 (4.4)	6 (6.1)	1.07 (0.92–1.24)	0.379	1.09 (0.97–1.23)	0.138
Viral load						
Suppressed or undetectable	542 (85)	90 (91)	Ref		Ref	
Unsuppressed	99 (15)	9 (9.1)	1.07 (0.98–1.16)	0.123	1.09 (0.98–1.21)	0.115
Patient phone number available						
No	452 (71)	83 (84)	Ref		Ref	
Yes	189 (29)	16 (16)	1.09 (1.02–1.16)	0.011	1.10 (1.01–1.90)	0.026
Patient physical address available						
No	295 (46)	36 (36)	Ref			
Yes	346 (44)	63 (64)	0.95 (0.85–1.06)	0.357		
Next of kin phone number available						
No	526 (82)	86 (87)	Ref			
Yes	115 (18)	13 (13)	1.06 (0.92–1.22)	0.425		

cPR, crude prevalence ratio; aPR, adjusted prevalence ratio; Ref, reference category

LTFU, loss to follow-up; CI, confidence interval

## Discussion

We have shown that self-transfer is a major reason for LTFU of patients from public ART clinics in Sheema District, Southwestern Uganda, between 2017 and 2021. We were also able to successfully trace the majority (87%) of those LTFU. The prevalence of self-transfers was significantly higher among young adults aged 18–30 years, females and those with asymptomatic or mild disease (WHO clinical stage 1–2), compared to their counterparts. The most common reasons for self-transfers were relocation, lack of money for transport, and lack of awareness on the transfer process. Presence of a phone contact in the patient’s file was independently associated with successful tracing of the LTFU patients. Given the high prevalence of self-transfers, these data highlight the need to account for self-transfers among patients considered as LTFU, to accurately estimate retention in care.

The prevalence of self-transfers among patients LTFU in the current study (76%) is higher than what other previous studies conducted in Uganda have reported, which ranged from 34 to 43% [5, 10]. Similarly, other studies, including systematic reviews conducted in low-and-middle-income countries (LMICs) have reported much lower estimates of self-transfers among patients considered LTFU, ranging from 12 to 54% [15–17]. This study finding is not surprising, and could be explained by the fact that our study was conducted in the era where the “treat all” guidelines had been rolled out. Moreover, it was previously hypothesized that most of the losses from ART clinics after the first year of the “treat all” guidelines' implementation would be unreported transfers to new ART-providing sites [6]. The increasing prevalence of self-transfers calls for the need to reorient ART services, to adapt to the evolving challenges in the ART care. Uganda is currently implementing differentiated service delivery (DSD) models. The DSD models adopt more patient-centered approaches, including switching to home-based care settings, reducing frequency of clinic visits, co-opting non-physician workers such as “expert clients”, and considering pharmacy-only refills for stable patients [18, 19]. Accelerating the implementation of such DSD models may reduce the increasing numbers that are self-transferring between ART-providing clinics. However, further research on the most appropriate, and cost-effective DSD models for the self-transfers in the various resource-limited settings, in the era of “treat all” are required.

In this study, participants with asymptomatic or mild disease, females and young adults aged 18–30 years were more likely to self-transfer, compared to their counterparts. Since the rollout of the “treat all” guidelines, PLHIV who begin treatment are increasingly asymptomatic or have mild disease. It was hypothesized that these would present new challenges to retention in care, including self-transfers [6]. Additionally, socio-demographic characteristics, including sex and age have been found to influence mobility of patients between ART-providing clinics [14]. Younger patients are more likely to relocate if they are not in school because of conflicting work schedules, and may be more economically disadvantaged compared to their older counterparts [20, 21]. Moreover, in the current study, the most common reasons for self-transferring were relocation, and lack of money for transport. These reasons have been cited in a number of studies done in similar resource-limited settings, as major contributors to loss to follow-up [5, 22, 23]. These findings imply that multifaceted interventions are required to minimize loss to follow-up. First and foremost, patient mobility is likely to become more common as the number of ART-providing sites increases, and healthcare workers should make deliberate efforts to raise patients’ knowledge and awareness of transfer procedures [14]. Secondly, providing incentives to patients such as drug supplies for a longer time could minimize frequent clinic visits and reduce transportation costs resulting in better retention in care [14, 18, 23]. Additionally, ART-providing sites should be ‘transfer-friendly’, assessing patients for their intention to transfer to other ART- providing sites so that they are appropriately supported through transfer decisions for better treatment outcomes [14].

Patients who had phone contacts available in their files were more likely to be successfully traced compared to others. This agrees with previous findings from studies done in Malawi and Ethiopia [24, 25]. Mobile phone technology has been suggested as one of the interventions to minimise loss to follow-up in LMICs [26]. The increasing availability of mobile phones, even in resource-limited settings provides an opportunity for successful tracing patients considered LTFU, so that their true outcomes are determined. On the basis of this finding, patients’ records in ART clinics should regularly have phone contacts updated at each visit, to ease subsequent tracing. Moreover, phone tracing could reduce the proportion of tracing patients via other resource- and labor-intensive methods, including field tracing.

Overall, our findings highlight the need to account for self-transfers among patients considered as LTFU, given the increasing trend of mobility and self-transfers. Furthermore, there is a need to implement efficient tracking systems such as electronic medical records that use unique patient identifiers to identify such self-transfers across other ART-providing facilities. Our findings also highlight a need to make deliberate efforts to maintain updated patient phone contacts in their files, to ease subsequent tracing. Given the high prevalence of self-transfers in this study, we recommend that future longitudinal studies assess outcomes among this population of self-transfers in similar resource-limited settings.

The main limitation of our study is that it was conducted in selected government-owned health facilities, in a rural district, in Southwestern Uganda. Thus, they may not be generalized to other settings, that are urban and in private health facilities. More studies in diverse settings, including private health facilities and outside Southwestern Uganda should be undertaken to corroborate our findings.

## Conclusions

Self-transfers accounted for the majority of patients recorded as LTFU. Successful tracing of patients recorded as LTFU should be carried out in order to accurately estimate retention in care. HIV clinics should regularly update contact information of patients to enable successful tracing when they may become LTFU.

## Data Availability

The datasets generated and analyzed for this study can be availed upon reasonable request from the corresponding author.

## Abbreviations

aPR: Adjusted prevalence ratio
ART: Anti-retroviral therapy
CI: Confidence interval
cPR: Crude prevalence ratio
DSD: Differentiated service delivery
HIV: Human immunodeficiency virus
LMICs: Low-and-middle-income countries
LTFU: Lost to follow-up
PLHIV: People living with HIV
SSA: Sub-Saharan Africa
WHO: World Health Organization
